# Laparoscopic segmental resection for tumours of the Angle of Treitz: a challenging but feasible surgical option. Results from a retrospective case-series analysis

**DOI:** 10.1007/s13304-020-00910-7

**Published:** 2020-11-04

**Authors:** Umberto Bracale, Emanuele Pontecorvi, Vania Silvestri, Diego Cuccurullo, Michele D’Ambra, Ruggero Lionetti, Andrea Coppola, Filippo Carannante, Felice Pirozzi, Roberto Peltrini, Antonio Sciuto, Francesco Corcione

**Affiliations:** 1 Department of General and Oncological Minimally Invasive Surgery, University Federico II of Naples, Via Pansini 5 7th Building, Naples, Italy; 2 Department of General Surgery, Azienda Ospedaliera Dei Colli, Monaldi Hospital, Naples, Italy; 3 Department of Geriatric Surgery, Università Campus Bio-Medico, Via Alvaro del Portillo 21, 00128 Rome, Italy; 4 Department of General Surgery, Santa Maria delle Grazie Hospital, Pozzuoli, Italy

**Keywords:** Duodenal cancer, Angle of Treitz, Laparoscopy, Overall survival, GIST, Duodenal segmental resection

## Abstract

Tumours of the small intestine are rare and account for about 5% of gastrointestinal tract neoplasms. The angle of Treitz (AT) could be defined as the intestinal loop comprised between the third duodenal portion and the first 10 cm of jejunum. A gold standard surgical treatment for AT neoplasm has not yet been well defined. This paper is focused on a very rare disease and at the best of our knowledge this is the largest case series in the literature about the Laparoscopic Segmental Resection (LSR) of AT tumours. Using a prospectively collected database, all data of consecutive patients, from January 2007 to May 2019, who underwent LSR for AT tumours at two different institutions were analysed**.** Patients’ demographics, intra and post-operative data, 30-day mortality and overall survival were collected. A total of 16 patients were retrieved from our database. The mean operative time was 206,5 ± 79 min. Conversion to open surgery was needed in two cases due to tumor size and, respectively, invasion of the transverse colon which required a multivisceral resection. The mean distal and proximal resection margins were 7.4 ± 2.2 and 3.9 ± 1.2 cm. The median number of harvested nodes was 9 ± 3. Pathological diagnosis was GIST in 11 cases, adenocarcinoma in 4 and sarcoma in 1 case. In conclusion, in experienced hands, LSR appears to be a safe and effective treatment option for tumours of the AT. Prospective studies are needed to confirm these findings.

**Electronic supplementary material:**

The online version of this article (10.1007/s13304-020-00910-7) contains supplementary material, which is available to authorized users.

## Introduction

Tumours of the small intestine are rare and account for about 5% of gastrointestinal tract neoplasms [1]. Both gastrointestinal stromal tumour (GIST) and adenocarcinoma (ADC) of the angle of Treitz (AT) are even rarer. The AT could be defined as the intestinal loop comprised between the third duodenal portion and the proximal 10 cm of the first jejunal loop [2, 3]. The standard treatment for AT tumours has not yet been well defined. Both segmental resection (SR) and pancreaticoduodenectomy (PD) have been proposed. Few case reports and one case series suggest an open SR as the treatment of choice, associated or not with a multi-visceral resection [3–6]. However, there is a great variability in the type of resection and subsequent reconstruction among the studies. Furthermore, despite laparoscopic approach has gained wide acceptance among surgeons [7], laparoscopic segmental resection (LSR) for AT tumours has been rarely described in the literature [8, 9]. Herein, we report our case series of AT tumours treated by LSR to evaluate its safety and feasibility. Our standardised surgical technique as well as long-term results are also reported and at the best of our knowledge this is the largest case series in literature about the Laparoscopic Segmental Resection (LSR) of AT tumours.

## Materials and methods

Internal ethical committee approval was obtained for data review. Using a prospectively collected database, all data of consecutive patients who underwent laparoscopic surgery for AT tumours from January 2007 to May 2019 at two different institutions (Department of General Surgery, Monaldi Hospital, Naples; Department of Gastroenterology, Endocrinology and Surgical Endoscopy, University Federico II of Naples) were retrospectively reviewed. The AT tract was defined as the intestinal loop comprised between the third duodenal portion and the proximal 10 cm of the first jejunal loop [2, 3].

Patient demographics, the American Society of Anaesthesiologists (ASA) score, previous abdominal surgery, operative time, intraoperative complications, conversion rate, pathologic outcomes (harvested lymph nodes, tumour size, length of specimen, staging according to Wittekid et al. [10]), postoperative complications according to the Clavien–Dindo classification [11], mortality, time to first flatus, length of hospital stay and readmission rate as well as long-term survival were collected.

All patients underwent a preoperative duodenoscopy with biopsy and neoplasm tattooing (if indicated) and full-body CT scan. In case of submucosal neoplasm, endoscopic ultrasound was performed.

Anastomotic leakage was considered as all conditions with clinical or radiologic features of anastomotic dehiscence in accordance with the UK Surgical Infection study Group [12, 13]. Discharge criteria included tolerance of oral intake, absence of nausea or vomiting, return of bowel function, absence of abdominal distention, no evidence of complications, adequate mobility, and patient acceptance [14].

The short-term follow-up included the first 30 postoperative days, while all adverse events that occurred later than the thirtieth day after surgery were considered as late complications. Each patient was followed-up every 6 months during the first two years and annually thereafter. Full body computed tomography was performed every 6 months for the first year and every year thereafter. After the surgical procedure, each patient was referred to an oncologist for further evaluation and treatment.

### Statistical analysis

Statistical analysis was carried out using IBM SPSS Statistics 25. Continuous data are expressed as mean ± standard deviation (SD). Categorical variables are expressed as frequencies and percentage. Estimated survival was evaluated with Kaplan–Meier methods.

### Surgical technique

The patient was placed in a supine reverse Trendelenburg position (30°) with right flank rotation and legs apart. Pneumoperitoneum was established with an open Veress-assisted technique and four trocars were placed in the upper abdomen. Following abdominal exploration, the gastrocolic ligament was divided to enter the lesser sac. Then, a Kocher manoeuvre was performed, and the superior mesenteric vessels were identified. The first jejunal loop was resected using an endoscopic linear stapler with a vascular cartridge at least 10 cm distal to the Treitz ligament. Then, the third and fourth duodenal portions were mobilized dissecting from the superior mesenteric vessels. In case of malignancy, lymph node dissection along the superior mesenteric artery was performed together with ligation of the inferior pancreaticoduodenal artery and first jejunal artery. The duodenum was divided between the second and the third portion, at least 2 or 5 cm above the neoplasm [3] for GIST and adenocarcinoma, respectively; an endoscopic linear stapler with a vascular cartridge was used. An intracorporeal mechanical side-to-side isoperistaltic duodenojejunostomy was performed between the second duodenal portion and the jejunum [9]. Stapler access enterotomy was closed with a double-layer absorbable running suture [15, 16]. A methylene blue dye leak test was performed to assess anastomotic integrity. The specimen was retrieved through a Pfannenstiel incision. Fascial defects ≥ 10 mm were closed [17]. The laparoscopic segmental resection technique is demonstrated in the video (Online Resource 1).

## Results

A total of 16 patients were retrieved from our database. Biometric features are reported in Table 1.

**Table 1:** Biometric features

	Patients no. 16
Sex (M/F)	8/8
Age (mean ± SD) years	60.37 ± 21.42
BMI (mean ± SD) kg/m^2^	25.27 ± 4.05
ASA score	
I no (%)	0
II no (%)	8 (50%)
III no (%)	8 (43.75%)
IV no (%)	0
Previous abdominal surgery no (%)	10 (32.5%)
Open appendectomy no (%)	4 (25%)
Laparoscopic cholecystectomy no (%)	3 (18.75%)
Open hysterectomy no (%)	2 (12.5%)
Laparoscopic left colectomy no (%)	1 (6.25%)
Preoperative symptoms	
Anemia no (%)	11 (68.75%)
Rectal bleeding/melena no (%)	2 (12.5%)
Nausea and vomiting no (%)	3 (18.75%)
Weight loss no (%)	3 (18.75%)
Bowel obstruction no (%)	1 (6.25%)
Abdominal pain no (%)	2 (12.5%)

*BMI* body mass index, *ASA* American society of anesthesiologists

Anaemia was the most frequent preoperative symptom. Intraoperative and postoperative data are reported in Table 2. Mean operative time was 206.5 ± 79 min. Intraoperative duodenoscopy was performed in two patients to assess the proximal resection margin and distance from the papilla of Vater. Conversion was needed in two cases due to large tumour size (15 cm) and infiltration of the transverse colon, respectively; an open multi-visceral resection was needed in the latter case. Two intraoperative complications occurred including a haemorrhage from the splenic vein, which needed a distal spleno-pancreatectomy, and an ileal loop injury that was sutured. All intraoperative complications were treated without conversion.

**Table 2:** Intra and post-operative data

Intra and post-operative data	
Operative time (mean ± SD) min	206,2 ± 79,8
Conversions no (%)	2 (12.5%)
Trasverse colon infiltration no (%)	1 (6.25%)
Tumor Dimension no (%)	1 (6.25%)
Intra operative complications no (%)	2 (12.5%)
Splenic vein injury no (%)	1 (6.25%)
Small Bowel injury no (%)	1 (6.25%)
Surgical laparoscopic associate procedures no (%)	2 (12.5%)
Trasverse colon resection no (%)	1 (6.25%)
Spleno-pancreasectomy no (%)	1 (6.25%)
Post-operative complications no (%)	2 (12.5%)
Clavien-Dindo classification	
II no (%)	1 (6.25%)
IV no (%)	1 (6.25%)
Time to flatus (mean ± SD) days	2.9 ± 0.9
Length of Stay (mean ± SD) days	9,1 ± 2,8
Histology	
GIST no (%)	11(68.75%)
Adenocarcinoma no (%)	4 (25%)
Sarcoma no (%)	1 (6.25%)
Size of neoplasm (mean ± SD) cm	9,1 ± 2,7
Nodes Harvested (median ± IQR) no	9 ± 3
Distance of tumour from distal margin (mean ± SD) cm	11,06 ± 3,08
Distance of tumour from proximal margin (mean ± SD) cm	3,8 ± 1,2

*GIST* gastro-intestinal stromal tumor

In two cases multi-visceral resection due to advanced disease was performed laparoscopically, which included distal spleno-pancreatectomy in the former and transverse colon resection in the latter.

The mean length of hospital stay was 9.1 ± 2.77 days. Two postoperative complications were found including one atrial fibrillation (Clavien–Dindo II) and one pulmonary embolism requiring intensive care unit management (Clavien–Dindo IV).

The mean distal and proximal resection margins were 7.4 ± 2.2 and 3.9 ± 1.2 cm. The median number of harvested nodes was 9 ± 3. Pathological examination showed a GIST in eleven cases, adenocarcinoma in four cases and one sarcoma (Table 3). After a mean follow-up of 51 ± 38 months, we found an estimated survival rate of 46.2%. Six deaths occurred: two patients died for disease recurrence, while four patients died for non-tumour-related causes (Table 3). The two recurrences were observed in the patient affected by sarcoma and in a patient affected by a GIST with a Ki67 index of 45% and a mitotic count of 6/50 HPF. Both patients developed local recurrence with infiltration of the pancreas and transverse colon.

**Table 3:** Clinical and pathologic characteristics of patients with tumors of the Angle of Treitz

Patient	Age	Sex	Size (cm)	Pathology	Nodes Harvested	Metastatic nodes	Distance from proximal margin	Distance from distal margin	Conversion	TNM	Grading	Mitotic rate	Multi-visceral resection	Site of progression	Survival in months	Causes of death
1	77	F	2	GIST	7	0	4	12	No	T1N0M0	G2	Low	No	None	118	Heart failure
2	15	M	5	GIST	12	0	3	14	No	T2N0M0	G2	High	No	None	114	None
3	48	M	9	GIST	9	0	3	10	No	T3N0M0	G2	High	Yes (Spleno-pancreasectomy)	Local recurrence with transverse colon invasion at 54 months follow-up	70	Disease recurrence
4	61	F	3,5	Adenocarcinoma	12	0	5,4	7	No	T2N0M0	G1	–	No	None	66	Heart failure
5	19	M	2	GIST	8	0	4,7	12	Yes (Transverse colon infiltration)	T1N0M0	G1	Low	Yes	None	95	None
6	33	F	5	Adenocarcinoma	14	0	5,2	14	No	T3N0M0	G2	–	No	None	92	None
7	80	F	4	GIST	8	0	3	9	No	T2N0M0	G2	Low	No	None	34	None
8	67	F	3,5	Sarcoma	11	1	4	12	No	T2N1M1	G1	–	Yes (Transverse Colon resection)	Local recurrence with pancreas invasion at 52 months follow-up	60	Disease recurrence
9	75	M	2	Adenocarcinoma	14	5	6	10	No	T3M2N0	G1	–	No	None	48	COPD complications
10	65	F	2,3	GIST	10	0	4,5	17	No	T2N0M0	G2	High	No	None	38	None
11	83	F	3.2	Adenocarcinoma	16	0	5,6	14	No	T1N0M0	G2	–	No	None	9	Pulmonary embolism
12	56	M	5	GIST	12	0	3,5	4	No	T2N0M0	G2	Low	No	None	12	None
13	74	M	5	GIST	14	0	4	10	No	T2N0M0	G1	High	No	None	27	None
14	77	M	5	GIST	12	0	3	11	No	T2N0M0	G2	High	No	None	15	None
15	59	M	5	GIST	14	0	2	12	No	T2N0M0	G2	Low	No	None	13	None
16	77	F	15	GIST	13	3	2	9	Yes (Dimension of tumour)	T4N1M0	G2	High	No	None	12	None

*GIST* gastro-intestinal stromal tumor, *COPD* chronic obstructive pulmonary disease

## Discussion

AT tumours pose a unique challenge for both identification of the tumour location and for surgical planning as well as treatment [18]. Preoperative diagnosis by conventional endoscopy may be difficult as the duodenojejunal region is not easily reached. Although not used in the present series, new modalities such as double-balloon enteroscopy or capsule endoscopy can make diagnosis of AT tumours easier [3]. Contrast-enhanced abdominal CT gives useful information regarding the location and the anatomical relationships between the mass and the surrounding structures, including vascular infiltration and invasion of adjacent organs. Biopsy is accepted as gold standard for diagnosing gastrointestinal tumours and it could be useful to properly plan a surgical approach with radical intent. However, obtaining a histological diagnosis of AT tumours preoperatively may be challenging due to (1) the difficulty in reaching these tumours, (2) major vessels in the surrounding that hamper approach under imaging guidance and (3) the possible submucosal location as in the cases of GISTs [3].

Due to their rarity and the variety of histotypes, the surgical treatment of AT tumours is not yet well defined. As reported in Table 4, only seven case reports and one case series of 13 patients (with a laparotomic approach) have been published on this topic since 1951 [3–6, 19–22]. Other cases were reported in the context of larger series concerning the treatment of different duodenal tracts [23, 24].

**Table 4:** Literature review about open approach

Author	Year	Type of article	Number of patients	Approach	Complications	Histology
Xie [3]	2014	Retrospective case series	13	Open	Not reported	13 GIST
Caruso [4]	2015	Case report	1	Open	None	GIST
Sista [5]	2012	Case report	1	Open	None	Adenocarcinoma
Fronticelli [6]	1996	Case report	1	Open	None	Adenocarcinoma
Nakano [20]	2013	Case report	1	Open	None	Adenocarcinoma
Markogiannakis [21]	2008	Case report	1	Open	None	Adenocarcinoma
Baig [22]	2011	Case report	1	First laparoscopic exploration then conversion to open	None	Adenocarcinoma
Bandi [23]	2015	Case report	1	Open	None	Adenocarcinoma

Surgical resection with negative margins and no intraoperative tumour spillage is accepted as treatment of choice for GISTs without the need of regional lymphadenectomy. Thus, SR is an option for tumours not amenable to wedge resection [25, 26].

PD with regional lymph node dissection has been initially suggested as a standard treatment for AT adenocarcinoma [27, 28]. Given the high morbidity rate of PD, a SR including the third and fourth duodenal portions and at least 10 cm of the first jejunal loop has been proposed [4, 5, 27, 29–32]. Indeed, similar mortality and morbidity rates between PD and SR have been reported by some authors [31–33]. Kaklamanos et al. [31] reported a morbidity rate of 27% for PD compared to 18% for SRD, with a mortality rate of 3% and, respectively, 1%. However, it must be acknowledged that a higher mortality rate is expected for PD when the procedure is not carried out in centres of excellence. Han et al. reported an increase from 3% up to 13.8–16.5% in those centres performing less than 5 PD per year [29]. Moreover, pancreatic leakage is absent or extremely rare following SRD. Surgical resection with negative margins and no intraoperative tumour spillage is accepted as treatment of choice for GISTs without the need of regional lymphadenectomy. Thus, SR is an option for tumours not amenable to wedge resection [25, 26]. While the type of operation would not affect long-term survival, this may be influenced by other factors including R1 or palliative resection, a locally advanced tumour, positive regional lymph nodes, and poor response to adjuvant chemotherapy [34]. Also, cancer location seems to affect survival, since tumours of the proximal duodenum show a worse prognosis than those arising in the third or fourth duodenal portions. This may be due to the closer relationship with the surrounding organs, which may be affected early in the course of the disease, as well as to the different routes of lymphatic drainage. Based on these considerations, PD may be recommended for tumours located in the proximal duodenum, while SR seems to be appropriate for distal tumours [30].

Regardless of the extent of resection, the importance of an adequate lymphadenectomy cannot be underscored [35]. Although the number of harvested lymph nodes for accurate N staging is debated —ranging from 6 to 15—[36–38], this number alone may not be a surrogate for adequate lymphadenectomy [39]. Tumours of the distal duodenum commonly involve the pancreaticoduodenal (#13) and superior mesenteric (#14) lymph node stations, while metastases to the pyloric (#5/6) and hepatic (#8 and #12) stations are usually not observed. Thus, the former lymph node stations should be included when SR for AT adenocarcinoma is performed [39].

Despite the limited number of patients and the histological heterogeneity of the disease treated, we found an estimated survival rate of 46.2% after a median follow-up of 51 ± 38 months. Due to the rarity of this tumour location, it would be very difficult to make considerations about the survival considering only the biology of histotype.

Surgery for AT tumours is technically challenging because of the variability and anatomical complexity of the duodenojejunal junction, which make it difficult to expose the operation field and visualize the intestine circumferentially [9]. Therefore, laparoscopy does not yet represent a standard approach for resection of AT tumours [8]. This approach has at least two major challenges to surgeons. The first is achieving an adequate proximal resection margin. It has been suggested that R0 resection for GISTs and adenocarcinomas can be attained with a safe margin of 2–5 cm, respectively [3, 6]. In our series, the mean proximal resection margin (3.9 ± 1.2 cm) was adequate, thus providing an R0 resection in all cases (Table 3). Reconstruction is also a challenging step. Indeed, the short stump of bowel after resection of the tumour at the duodenojejunal junction makes it difficult to handle during the anastomosis [9]. Different reconstruction methods through the open approach have been reported such as hand-sewn end-to-end duodenojejunostomy [40] and mechanical end-to-side duodenojejunostomy [10]. Instead, the use of a linear stapler allows a minimally invasive reconstruction in a side-to-side fashion between the duodenal stump and the jejunum.

To overcome the hurdles of intestinal exposure and reconstruction at the duodenojejunal flexure, the intestinal derotation technique described by Valdoni has been proposed as a valid option for SR of the third and fourth duodenal portions [41]. After derotation, the duodenojejunal flexure becomes straight, thus allowing a simplified resection and reconstruction, similar to those of a jejunal loop. This complex procedure can be carried out with a minimally invasive approach by surgeons with extensive experience in both laparoscopic surgery and open intestinal derotation [42].

Laparoscopic SR remains a technically demanding procedure also for experienced surgeons. Indeed, two intraoperative complications including splenic vein injury and bowel injury occurred in our series. Moreover, conversion should be part of the surgical strategy to provide a complete resection or reconstruction based on intraoperative evaluation [43]. Despite the technical difficulties of laparoscopic approach, magnification of the image may allow a more accurate dissection and thus it could help in preventing injuries of the pancreas and mesenteric axis. Moreover, a totally laparoscopic procedure may be beneficial in terms of quick recovery and short hospital stay as well as reduced surgical site infections [8].

This paper has some limitations. First, the historical bias due to the long-time span in which the patients has been treated. Then, the retrospective nature of the study, the heterogeneity of the disease treated and the small sample size analysed. However, our paper is focused on a very rare disease and, at the best of our knowledge, this is the largest case series in the literature about LSR for tumours of the duodenojejunal flexure. A single retrospective study including eight patients undergoing LSR for a GIST has been published [9]. As in our series, all patients underwent curative resection with a similar postoperative morbidity (12.5%) and no mortality. Neither conversions to open surgery nor recurrences at 37-month follow-up were observed.

In conclusion, in experienced hands, laparoscopic segmental resection appears to be a safe and effective treatment option for tumours of the angle of Treitz. Larger prospective studies are needed to confirm these findings.

## Electronic supplementary material

Below is the link to the electronic supplementary material.

Supplementary file1 (MP4 256673 KB)

## References

[1] Moglia A, Menciassi A, Dario P, Cuschieri A (2007). Clinical update: endoscopy for small-bowel tumours. Lancet.

[2] Gray H (1918) Anatomy of the human body. Philadelphia: Lea & Febiger, 1918; Bartleby.com, 2000. www.bartleby.com/107/.

[3] Xie YB, Liu H, Cui L, Xing GS, Yang L, Sun YM, Bai XF, Zhao DB, Wang CF, Tian YT (2014). Tumors of the angle of Treitz: a single-center experience. World J Gastroenterol.

[4] Caruso F, Nencioni M, Zefelippo A, Rossi G, Caccamo L (2016). Is duodenojejunal anastomosis to the left of the superior mesenteric vessels a feasible option for tumors of the angle of Treitz?. Tumori.

[5] Sista F, Santis GD, Giuliani A, Cecilia EM, Piccione F, Lancione L, Leardi S, Amicucci G (2012). Adenocarcinoma of the third duodenal portion: case report and review of literature. World J Gastrointest Surg.

[6] Fronticelli CM, Borghi F, Gattolin A, Ferrero A, Delsedime L (1996). Primary adenocarcinoma of the angle of Treitz. Case report and review of the literature. Arch Surg (Chicago III 1960).

[7] Bracale U, Pacelli F, Milone M, Bracale UM, Sodo M, Merola G, Troiani T, Di Salvo E (2017). Laparoscopic treatment of abdominal unicentric castleman's disease: a case report and literature review. BMC Surg.

[8] Corcione F, Pirozzi F, Sciuto A, Galante F, Bracale U, Andreoli F (2013). Laparoscopic pancreas-preserving subtotal duodenectomy for gastrointestinal stromal tumor. Minim Invasive Ther Allied Technol.

[9] Tanaka E, Kim M, Lim JS, Choi YY, Saklani A, Noh SH, Hyung WJ (2015). Usefulness of laparoscopic side-to-side duodenojejunostomy for gastrointestinal stromal tumors located at the duodenojejunal junction. J Gastrointest Surg.

[10] Wittekind C, Oberschmid B (2010). TNM classification of malignant tumors 2010: General aspects and amendments in the general section. Pathologe.

[11] Clavien PA, Barkun J, de Oliveira ML, Vauthey JN, Dindo D, Schulick RD, de Santibanes E, Pekolj J, Slankamenac K, Bassi C, Graf R, Vonlanthen R, Padbury R, Cameron JL, Makuuchi M (2009). The Clavien-Dindo classification of surgical complications: five-year experience. Ann Surg.

[12] Rahbari NN, Weitz J, Hohenberger W, Heald RJ, Moran B, Ulrich A, Holm T, Wong WD, Tiret E, Moriya Y, Laurberg S, den Dulk M, van de Velde C, Buchler MW (2010). Definition and grading of anastomotic leakage following anterior resection of the rectum: a proposal by the international study group of rectal cancer. Surgery.

[13] Peel AL, Taylor EW (1991). Proposed definitions for the audit of postoperative infection: a discussion paper. Surgical infection study group. Ann R Coll Surg Engl.

[14] Bracale U, Azioni G, Rosati M, Barone M, Pignata G (2009). Deep pelvic endometriosis (Adamyan IV stage): multidisciplinary laparoscopic treatments. Acta Chir Iugosl.

[15] Reggio S, Sciuto A, Cuccurullo D, Pirozzi F, Esposito F, Cusano D, Corcione F (2015). Single-layer versus double-layer closure of the enterotomy in laparoscopic right hemicolectomy with intracorporeal anastomosis: a single-center study. Tech Coloproctol.

[16] Bracale U, Merola G, Cabras F, Andreuccetti J, Corcione F, Pignata G (2018). The use of barbed suture for intracorporeal mechanical anastomosis during a totally laparoscopic right colectomy: is it safe? A retrospective nonrandomized comparative multicenter study. Surg Innov.

[17] Crocetti D, Sapienza P, Pedulla G, De Toma G (2014). Reducing the risk of trocar site hernias. Ann R Coll Surg Engl.

[18] Biello AR, Lin-Hurtubise KM, Condon FJ, Allen EJ (2019). Duodenal adenocarcinoma at the ligament of Treitz: management and outcome. Hawaii J Health Soc Welf.

[19] Nakano T, Sugawara K, Hirau K, Hirano Y, Hashimoto M, Kaiho T, Ohuchi N (2013). Primary adenocarcinoma of the fourth portion of the duodenum: "a case report and literature review". Int J Surg Case Rep.

[20] Markogiannakis H, Theodorou D, Toutouzas KG, Gloustianou G, Katsaragakis S, Bramis I (2008). Adenocarcinoma of the third and fourth portion of the duodenum: a case report and review of the literature. Cases J.

[21] Baig SJ (2011). Malignant tumor at the fourth part of duodenum mimicking wilkie syndrome. Indian J Surg.

[22] Bandi M, Scagliarini L, Anania G, Pedriali M, Resta G (2015). Focus on the diagnostic problems of primary adenocarcinoma of the third and fourth portion of the duodenum. Case report G Chir.

[23] Meijer LL, Alberga AJ, de Bakker JK, van der Vliet HJ, Le Large TYS, van Grieken NCT, de Vries R, Daams F, Zonderhuis BM, Kazemier G (2018). Outcomes and treatment options for duodenal adenocarcinoma: a systematic review and meta-analysis. Ann Surg Oncol.

[24] Shen Z, Chen P, Du N, Khadaroo PA, Mao D, Gu L (2019). Pancreaticoduodenectomy versus limited resection for duodenal gastrointestinal stromal tumors: a systematic review and meta-analysis. BMC Surg.

[25] Cananzi FCM, Ruspi L, Sama L, Sicoli F, Gentile D, Minerva EM, Cozzaglio L, Quagliuolo V (2019). Short-term outcomes after duodenal surgery for mesenchymal tumors: a retrospective analysis from a single tertiary referral center. Updates Surg.

[26] Kumar AP, Swain SK, Das S, Paul S, Renganathan K, Zirpe D, Kumar G, Gopasety M, Radhakrishna P, Balachandar TG (2015). Duodenojejunal flexure tumors: surgical difficulties with case series. J Gastrointest Oncol.

[27] Ito H, Perez A, Brooks DC, Osteen RT, Zinner MJ, Moore FD, Ashley SW, Whang EE (2003). Surgical treatment of small bowel cancer: a 20-year single institution experience. J Gastrointest Surg Off J Soc Surg Aliment Tract.

[28] Cheng XD, Du YA, Xu ZY, Huang L, Yang LT, Wang B, Zhou YM, Yu PF, Yu QM (2012). A modified pancreaticojejunostomy: kissing pancreaticojejunostomy. Hepatogastroenterology.

[29] Han SL, Cheng J, Zhou HZ, Zeng QQ, Lan SH (2010). The surgical treatment and outcome for primary duodenal adenocarcinoma. J Gastrointest Cancer.

[30] Czaykowski P, Hui D (2007). Chemotherapy in small bowel adenocarcinoma: 10-year experience of the British Columbia Cancer Agency. Clin Oncol (R Coll Radiol).

[31] Kaklamanos IG, Bathe OF, Franceschi D, Camarda C, Levi J, Livingstone AS (2000). Extent of resection in the management of duodenal adenocarcinoma. Am J Surg.

[32] Chung RS, Church JM, vanStolk R (1995). Pancreas-sparing duodenectomy: indications, surgical technique, and results. Surgery.

[33] de Castro SM, van Eijck CH, Rutten JP, Dejong CH, van Goor H, Busch OR, Gouma DJ (2008). Pancreas-preserving total duodenectomy versus standard pancreatoduodenectomy for patients with familial adenomatous polyposis and polyps in the duodenum. Br J Surg.

[34] Agrawal S, McCarron EC, Gibbs JF, Nava HR, Wilding GE, Rajput A (2007). Surgical management and outcome in primary adenocarcinoma of the small bowel. Ann Surg Oncol.

[35] Cloyd JM, George E, Visser BC (2016). Duodenal adenocarcinoma: advances in diagnosis and surgical management. World J Gastrointest Surg.

[36] Poultsides GA, Huang LC, Cameron JL, Tuli R, Lan L, Hruban RH, Pawlik TM, Herman JM, Edil BH, Ahuja N, Choti MA, Wolfgang CL, Schulick RD (2012). Duodenal adenocarcinoma: clinicopathologic analysis and implications for treatment. Ann Surg Oncol.

[37] Gibbs JF (2004). Duodenal adenocarcinoma: is total lymph node sampling predictive of outcome?. Ann Surg Oncol.

[38] Compton CC, Byrd DR, Garcia-Aguilar J, Kurtzman SH, Olawaiye A, Washington MK (2012). AJCC cancer staging atlas, a companion to the seventh editions of the AJCC cancer staging manual and handbook. Springer Verlag NY.

[39] Sakamoto T, Saiura A, Ono Y, Mise Y, Inoue Y, Ishizawa T, Takahashi Y, Ito H (2017). Optimal lymphadenectomy for duodenal adenocarcinoma: does the number alone matter?. Ann Surg Oncol.

[40] Chung JC, Kim HC, Chu CW (2011). Segmental duodenectomy with duodenojejunostomy of gastrointestinal stromal tumor involving the duodenum. J Korean Surg Soc.

[41] De Nicola P, Di Bartolomeo N, Francomano F, D'Aulerio A, Innocenti P (2005). Segmental resection of the third and fourth portions of the duodenum after intestinal derotation for a GIST: a case report. Suppl Tumori.

[42] Valle M, Federici O, Tarantino E, Corona F, Garofalo A (2009). Laparoscopic intestinal derotation: original technique. Surg Laparosc Endosc Percutan Tech.

[43] Leon P, Iovino MG, Giudici F, Sciuto A, de Manzini N, Cuccurullo D, Corcione F (2018). Oncologic outcomes following laparoscopic colon cancer resection for T4 lesions: a case-control analysis of 7-years' experience. Surg Endosc.

